# Cis-Acting Chaperoning by Macromolecular Tethering: A Built-In Layer of Cellular Chaperoning

**DOI:** 10.3390/ijms27083360

**Published:** 2026-04-09

**Authors:** Seong Il Choi, Yoontae Jin, Yura Choi, Baik L. Seong

**Affiliations:** 1 Department of Pediatrics, Severance Hospital, Institute of Allergy, Brain Korea 21 PLUS Project for Medical Science, Yonsei University College of Medicine, Seoul 03722, Republic of Korea; 2 Vaccine Innovative Technology ALliance (VITAL)-Korea, Seoul 03722, Republic of Korea; yoontae_jin@yonsei.ac.kr (Y.J.); yurachoi@yonsei.ac.kr (Y.C.); 3 Department of Microbiology and Immunology, Institute for Immunology and Immunological Diseases, Graduate School of Medical Science, Brain Korea 21 Project, Yonsei University College of Medicine, Seoul 03722, Republic of Korea; 4 Department of Integrative Biotechnology, Yonsei University, Incheon 21983, Republic of Korea; 5 Department of Microbiology, College of Medicine, Yonsei University, Seoul 03722, Republic of Korea

**Keywords:** protein folding, aggregation, macromolecular tethering, cellular chaperoning, colloidal stability, excluded volume, charge, molecular chaperone

## Abstract

The molecular chaperone paradigm has shaped modern views of assisted protein folding, yet it does not fully capture the physical context in which *de novo* folding occurs in cells. A defining feature of the cellular milieu is macromolecular tethering in cis, whereby nascent polypeptides remain physically linked—through covalent or persistent associations—to ribosomes, lipid bilayers, or pre-folded domains of multidomain proteins. Because molecular chaperones have traditionally been defined as reversible binders acting in trans, this cis-acting mode has remained conceptually underappreciated. Cellular macromolecules, by virtue of their steric bulk and surface charges, can suppress aggregation of tethered polypeptides, thereby increasing productive folding yield. By analogy to colloidal stability, this repulsion-mediated control of aggregation suggests that cellular macromolecules can exhibit intrinsic chaperone-like activity largely independent of whether the linkage occurs in cis or in trans. This property provides a conceptual basis for linking cis- and trans-acting chaperoning. Thus, macromolecular tethering in cis may constitute a built-in layer of cellular chaperoning, distinct in physical linkage yet mechanistically related to conventional molecular chaperones.

## 1. Introduction

Understanding how proteins efficiently attain their functional conformations within the cellular environment remains a central question in molecular biology [1,2]. Anfinsen’s thermodynamic hypothesis states that the native structure of a protein is determined by its amino acid sequence and represents the most thermodynamically stable conformation under physiological conditions [3]. This principle of spontaneous folding was derived primarily from in vitro studies of small, single-domain proteins under dilute conditions [4]. In cells, however, folding proceeds in a crowded, heterogeneous, and highly interactive milieu in which polypeptides are continuously exposed to misfolding and aggregation. Cellular factors such as molecular chaperones therefore play critical roles in ensuring efficient protein folding and maintaining proteome integrity [5].

The discovery of molecular chaperones in the late 1980s led to the emergence of the modern paradigm of assisted protein folding [6,7,8]. Molecular chaperones, including GroEL/ES, Hsp70, and Hsp90, are classically defined as proteins that facilitate the folding of other proteins without becoming part of their final structures [9]. They typically act in trans by transiently binding exposed hydrophobic segments of non-native polypeptides through reversible, often ATP-driven cycles, thereby playing central roles in protein quality control and proteostasis [5,10]. Molecular chaperones function as genuine folding catalysts in specific contexts and also frequently assist folding indirectly by suppressing aggregation, sometimes at the expense of folding rate [11,12,13,14,15,16,17]. It is therefore important to distinguish between direct folding assistance, which catalyzes intramolecular folding, and indirect folding assistance, which increases folding yield by suppressing intermolecular aggregation [18].

**Figure 1:**
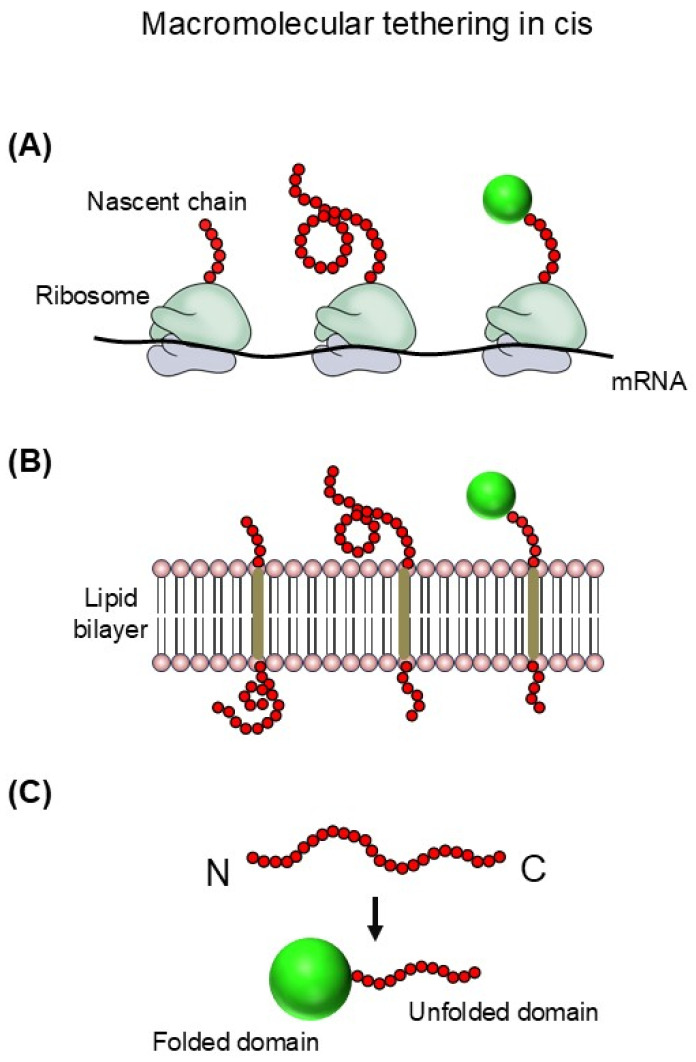
Macromolecular tethering in cis. Representative forms of cis-acting macromolecular tethering. (**A**) Ribosome tethering, (**B**) Membrane tethering, and (**C**) Domain tethering. In each case, the polypeptide remains covalently or stably linked to a cellular macromolecule. Aggregation-prone polypeptide segments are shown in red.

In cells, nascent polypeptides are physically tethered in cis to macromolecular scaffolds such as ribosomes (for all newly synthesized polypeptides), lipid bilayers (~30% of the proteome), or domains within multidomain proteins (~70% of the proteome), rather than existing as freely diffusing species in solution, as illustrated in Figure 1 [19,20]. These tethering linkages in cis are either covalent or otherwise persistent, in contrast to the transient and reversible interactions characteristic of trans-acting molecular chaperones. Yet, despite being an intrinsic feature of *de novo* folding and pervasive across the proteome, macromolecular tethering has received little attention in discussions of cellular chaperoning. Notably, cis-linkage-based strategies such as ribosome display, membrane anchoring, and fusion to soluble partners are widely used in biotechnology to improve protein solubility and folding yield [21]. However, these practical applications have rarely been considered physiologically relevant forms of chaperoning [21,22]. Mechanistically, it has been proposed that the steric bulk and surface charges of cellular macromolecules—including molecular chaperones—can generate repulsive forces that reduce aggregation of tethered polypeptides, thereby increasing productive folding yield [23]. Because these repulsive effects can depend on macromolecular size and surface properties, their influence on intermolecular aggregation scales with the size of the tethered macromolecule. Viewed in this light, principles analogous to colloidal stability can provide a coherent physicochemical basis for understanding cis-acting chaperoning by macromolecular tethering [22,23]. Such mechanisms, central to aggregation inhibition and proteome solubility, are not readily accommodated within traditional trans-acting molecular chaperone models that largely focus on hydrophobic interaction-mediated substrate recognition and stabilization. These considerations motivate a broader view of cellular chaperoning that explicitly incorporates cis-acting chaperoning by macromolecular tethering and connects cis- and trans-acting chaperoning within a unified framework.

In this review, we examine (i) the conceptual underappreciation of cis-acting chaperoning, (ii) repulsion-mediated aggregation inhibition as a mechanistic link between cis- and trans-acting chaperoning, (iii) ribosome tethering, domain tethering, and membrane tethering as representative forms of cis-acting chaperoning, and (iv) the interconvertibility of cis- and trans-acting chaperoning through changes in the mode of physical linkage. Together, these insights highlight cis-acting chaperoning by macromolecular tethering as a largely overlooked yet potentially important component of cellular chaperoning.

## 2. Cis-Acting Chaperoning: A Blind Spot of Modern Protein Science

**Figure 2:**
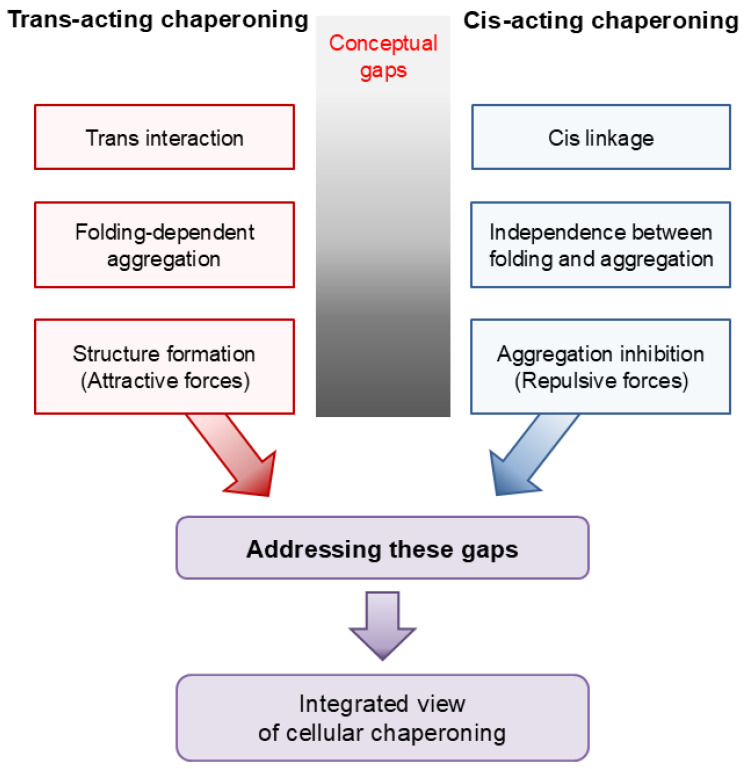
Conceptual gaps between prevailing frameworks in protein science and cis-acting chaperoning by macromolecular tethering. Schematic illustration of the differences between trans-acting chaperoning (**left**) and cis-acting chaperoning (**right**). Analysis of these gaps links cis- and trans-acting chaperoning, providing an integrated view of cellular chaperoning.

Three frameworks have been particularly influential in protein science: (i) a trans-acting interaction-centric framework, (ii) a folding-centric view of aggregation, and (iii) a structure-formation-centric framework rooted primarily in attractive interactions. The conventional definition and models of molecular chaperones are largely grounded in these frameworks. However, as illustrated in Figure 2, these prevailing frameworks do not explicitly incorporate cis-acting chaperoning by macromolecular tethering, creating conceptual gaps in our understanding of cellular chaperoning. In particular, the cis linkage of polypeptides to cellular macromolecules, the independence between intramolecular folding and intermolecular aggregation, and repulsion-mediated aggregation inhibition remain underemphasized within these frameworks. Addressing these gaps clarifies aspects of cellular chaperoning that are not fully captured in prevailing models and provides a conceptual link between cis- and trans-acting chaperoning. However, this link does not require expanding the definition of molecular chaperones to encompass cellular macromolecules with chaperone-like activity in cis; rather, it supports an expanded view of cellular chaperoning.

### 2.1. Trans-Acting Interaction-Centric Framework

By definition, molecular chaperones are not incorporated into their clients’ final structures and are therefore classified as trans-acting proteins [9]. Within this definition, chaperoning is typically described as involving transient and reversible protein–protein interactions [5,10]. As a result, cis-acting chaperoning mediated by macromolecular scaffolds such as ribosomes, membranes, and folded domains falls conceptually outside the conventional scope of this definition. Folding assistance by molecular chaperones has often been implicitly framed by analogy to enzymatic catalysis. Molecular chaperones bind transiently to non-native conformations and dissociate once folding is complete, much like enzymes that preferentially bind substrates but not products [24,25,26]. Within this framework, the ability to selectively interact with non-native states while avoiding stable association with the folded product has become a central feature of molecular chaperone function. Molecular chaperones can readily be compared with classical in vitro refolding systems and integrated into existing folding frameworks, because the initial reactants (the unfolded state) and final products (the folded state) are preserved as the same molecular species in both cases. Macromolecular tethering, by contrast, does not satisfy this selectivity-based criterion, as it links polypeptides irrespective of their folding state. Diverse chaperone-like molecules—including ribosomes, RNAs, DNAs, polyphosphates, and proteins such as Spy—have been identified [27,28,29,30,31]. In all cases, their activities have been investigated in trans-acting interaction-based systems. More broadly, protein science has historically tended to approach cellular interactions primarily through a trans-acting lens, as reflected in widely used methodologies such as yeast two-hybrid screening and interactome mapping [32,33,34].

Thermodynamic analyses of protein interactions are commonly based on equilibrium theory, which describes reversible binding between well-defined states (e.g., a protein P and an interacting partner M) [35,36]. Under this formalism, trans-acting interactions (P + M ⇌ PM) are characterized by an equilibrium constant, which reflects the balance between association and dissociation rates and the corresponding standard Gibbs free energy change. In contrast, cis-tethering (P + M → P − M) is not readily represented as a simple reversible binding equilibrium. For this reason, cis-tethering is not fully captured within the classical equilibrium formalism used for reversible binding interactions. However, from a physical standpoint, cis-tethering can be regarded as a kinetic limiting case of trans association in which the effective dissociation rate is negligible on the timescale of interest. Once a complex between P and M is formed, the central issue becomes how the attached macromolecule M influences protein folding and aggregation, irrespective of whether the association is maintained through cis or trans linkage. Accordingly, cis- and trans-acting chaperoning can be interpreted at the level of the complexed state and compared on a common physicochemical basis.

### 2.2. Protein Folding-Centric Aggregation Description

**Figure 3:**
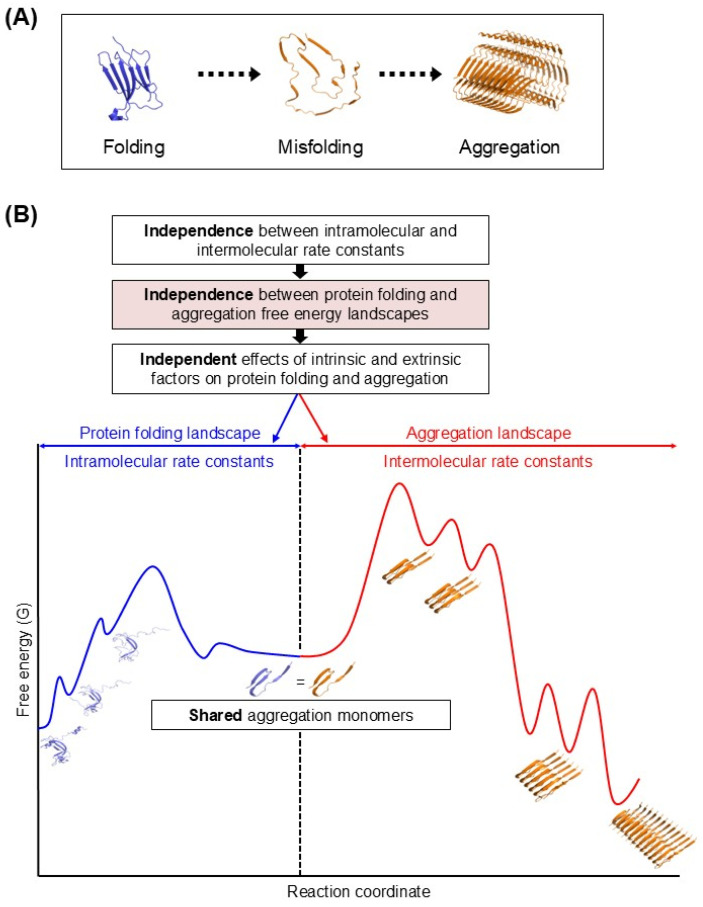
Relationship between protein folding and aggregation. (**A**) Traditional folding-centric view in which aggregation is treated as a downstream consequence of folding or misfolding along a single reaction pathway. (**B**) Modified view in which folding and aggregation are independent yet interconnected through a shared pool of aggregation monomers (adapted from Ref. [18]).

Proper folding and thermodynamic stability generally reduce aggregation propensity, whereas partially folded or misfolded conformations can act as aggregation precursors [37,38]. Aggregation has therefore often been viewed as a downstream consequence of folding or misfolding (Figure 3A), as exemplified by the Lumry–Eyring model [39]. In this folding-centric framework, folding, misfolding, and aggregation are frequently interpreted as occurring along a single reaction pathway. However, this view does not fully account for intermolecular aggregation processes and for indirect folding assistance mediated by aggregation inhibition. For instance, a protein encapsulated in the GroEL/ES chamber is protected from aggregation, regardless of its conformational state, indicating that aggregation suppression is not strictly determined by folding state; conversely, the chamber can simultaneously modulate intramolecular folding independently of its aggregation suppression effect [18,40]. These observations highlight the need for a more comprehensive framework for describing the folding–aggregation relationship. From a physical chemistry perspective, protein folding and aggregation constitute a coupled reaction system involving both intra- and intermolecular processes. In such systems, intramolecular and intermolecular reactions are governed by independent kinetic and thermodynamic parameters [41,42]. Accordingly, intramolecular folding (or misfolding) and intermolecular aggregation should be treated as independent processes that are nonetheless coupled through shared aggregation monomers (Figure 3B) [18]. Factors—such as macromolecular tethering and molecular chaperones—can therefore act independently on folding and aggregation. Both the final folding yield in the presence of aggregation and the extent of aggregation are determined by the combined yet independent effects of these factors on intra- and intermolecular reactions. Folding and misfolding influence aggregation primarily by modulating the concentration of shared aggregation monomers rather than by altering the intrinsic kinetic or thermodynamic parameters of aggregation. In this new view, the intermolecular aggregation process lies beyond the folding-centric framework and can be analyzed, at least in part, analogously to aggregation in small molecules, polymers, and colloidal systems. This perspective suggests that intermolecular protein aggregation can be interpreted by analogy to colloidal stability.

Classical in vitro denaturation–refolding experiments, which resemble post-translational folding in vivo, have long served as the dominant reference framework for protein folding, even though cotranslational folding on the ribosome is now widely recognized. Much of this work has focused on intramolecular folding of nascent chains, and the effects of ribosome tethering on aggregation have largely been inferred from its influence on folding, rather than examined independently, consistent with the folding-centric framework. Experimentally, dissecting these independent effects is challenging and differs substantially from conventional denaturation–refolding assays using denaturants such as urea. In tethered systems, the macromolecule must remain intact during unfolding, and its presence introduces background signals that complicate the quantitative assessment of folded and unfolded states. As a result, folding under tethered conditions has relied primarily on specialized techniques such as optical tweezers, single-molecule FRET, and isotope-labeled NMR spectroscopy [43,44,45]. However, these approaches are primarily designed to probe intramolecular folding and are not readily applicable to the analysis of multimolecular aggregation processes. In addition, aggregation often involves complex kinetics and long timescales, making it difficult to preserve tethered macromolecules throughout experiments, particularly under denaturing conditions. Intramolecular folding kinetics, thermodynamics, and structural features can be directly compared between free and tethered proteins, because folding is an intramolecular reaction. In contrast, intermolecular aggregation can produce fundamentally different structures in freely diffusing versus tethered systems, rendering aggregation kinetics, thermodynamics, and pathways not directly comparable between the two conditions. This asymmetry, together with the folding-centric framework and experimental challenges, helps explain the relative scarcity of systematic studies on aggregation under tethered conditions. By comparison, in biotechnology, the chaperone-like effects of macromolecular tethering are readily observed empirically, even in the absence of detailed mechanistic understanding [46,47].

The independence between folding and aggregation clarifies that direct and indirect folding assistance by molecular chaperones are mechanistically distinct [18]. Thus, emphasizing molecular chaperones solely as foldases can obscure their contribution to productive folding through aggregation prevention. Molecular chaperones often reduce the apparent folding rate by binding exposed hydrophobic regions of non-native states, which are normally buried in the native structure, thereby requiring cycles of substrate binding and release [14,48]. Small heat shock proteins prevent aggregation even while folding is arrested [49,50]. This behavior contrasts with classical folding catalysts, such as protein disulfide isomerase (PDI) and peptidyl–prolyl isomerases (PPI), which directly accelerate the rates of specific intramolecular rearrangements. In the absence of aggregation, misfolded states or kinetic traps at the intramolecular level can often be overcome by the protein’s intrinsic folding capacity. The apparent paradox—slower folding rates yet higher folding yields in the presence of aggregation—is resolved by recognizing that molecular chaperones modulate intramolecular folding and intermolecular aggregation independently [18]. Accordingly, the indirect folding assistance provided by molecular chaperones may share mechanistic similarities with cis-acting chaperoning by macromolecular tethering, as described below.

### 2.3. Structure Formation-Centric Framework

Protein structures are central to understanding biological function and underpin rational drug design. Accordingly, modern protein science has adopted a structure formation-centric framework, interpreting folding, binding, native assembly, and aggregation, such as amyloid fibril formation, primarily in terms of specific three-dimensional structures. In this view, structure formation is explained mainly by structure-stabilizing attractive interactions intrinsic to the polypeptide chain, including hydrophobic, van der Waals, hydrogen-bonding, and electrostatic interactions [51,52,53]. Consistently, hydrophobic interactions between molecular chaperones and their substrates are commonly invoked to explain substrate recognition and stabilization against aggregation [5,25]. However, this structure formation-centric framework can be misleading if its limitations are not carefully considered. For instance, aggregation inhibition (or solubility maintenance) represents an anti-structure-formation regime, in which aggregation-preventing forces—such as intermolecular repulsions and entropic effects—dominate over aggregation-stabilizing attractions under given conditions. Recognizing this regime highlights the importance of repulsion-mediated aggregation inhibition in understanding cellular chaperoning. Consistent with this view, colloid science describes aggregation inhibition primarily in terms of electrostatic and steric repulsions that prevent aggregate formation [54,55,56]. This reasoning suggests that cellular chaperoning can be understood by analogy to colloidal stability.

Charges play a central role in maintaining solubility and preventing aggregation through electrostatic repulsion and desolvation penalties, from small molecules and peptides to cellular macromolecules and colloidal particles [57,58,59]. Another example is steric shielding by the GroEL/ES chamber, in which a client protein is protected from intermolecular self-aggregation through physical exclusion, independent of attractive interactions with the chamber interior. In this case, the aggregation landscape of the client is dictated by the physical properties of the extrinsic chamber rather than by intrinsic properties of the protein. Aggregation inhibition mediated by electrostatic and steric repulsions is not fully captured within folding-centric, structure-formation-centric, and attractive-interaction-centric frameworks. These examples explain why structure-formation landscapes defined by intrinsic properties of proteins can diverge from those observed in vivo, where extrinsic macromolecular constraints and structure-destabilizing forces can predominate under certain conditions. Macromolecular tethering represents a prominent example of such divergence in the context of protein aggregation.

The cytosol-exposed nascent chains tethered to ribosomes are often implicitly assumed to exhibit aggregation behavior similar to that of free nascent chains, consistent with both attractive-interaction-centric and folding-centric frameworks. Under this assumption, aggregation propensity is effectively treated as being insensitive to the physical scale of the tethered cellular macromolecule. Ribosomes are megadalton-sized assemblies (∼2–4 MDa) with an average diameter of ~20–30 nm and carry several thousand negative charges, derived primarily from their rRNA components [60,61]. Moreover, aggregation is a concentration-dependent, multimolecular assembly process that exhibits a degree of specificity [38,39,41]. In multimolecular assembly, the magnitude of electrostatic and steric effects exerted by tethered macromolecules can depend on their size, such that as the effective radius (*r*) increases, the surface area and excluded volume scale geometrically with *r*^2^ and *r*^3^, respectively [62]. These scaling relationships underscore that aggregation control can scale with the size of the tethered macromolecule, providing a physical basis for interpreting cis-acting chaperoning by macromolecular tethering.

## 3. Repulsion-Based Principles Underlying Cellular Chaperoning

Indirect folding assistance primarily relies on aggregation inhibition, which is largely governed by intermolecular repulsive forces. Viewing cellular chaperoning through a repulsion-based lens can provide a common mechanistic basis for linking cis- and trans-acting chaperoning. Moreover, although the molecular chaperone field, colloid science, and biotechnology all address aggregation, their insights have rarely been considered together within a shared physical framework; repulsion-mediated aggregation inhibition offers a basis for such integration. Unlike attractive interactions found in final structures, repulsive interactions are inherently difficult to infer from them [63]. For this reason, the repulsion-based description discussed here is necessarily qualitative.

### 3.1. Cellular Macromolecules as Large Aggregation Gatekeepers

**Figure 4:**
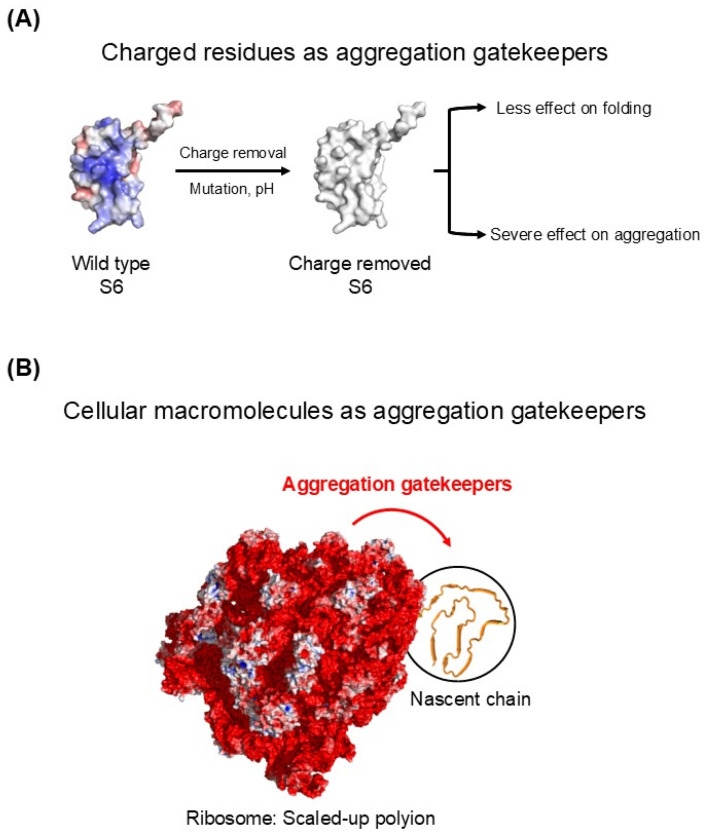
The aggregation gatekeeper concept from residues to macromolecules. (**A**) Removal of surface charges from ribosomal protein S6 has a minimal effect on cooperative folding but markedly increases aggregation, illustrating that charged residues can act as aggregation gatekeepers independently of folding. Negative and positive charges are shown in red and blue, respectively. (**B**) By extension, cellular macromolecules such as ribosomes can function as macromolecular-scale aggregation gatekeepers for their tethered polypeptides, suppressing intermolecular aggregation while allowing intrinsic folding to proceed.

Protein solubility has often been discussed in terms of colloidal stability [64,65,66,67,68,69]. A prominent example is provided by intrinsically disordered proteins or regions (IDPs/IDRs). Many IDPs/IDRs have low hydrophobic content and high net charge, features that can promote solubility [70]. Similarly, globular proteins maintain solubility through surface properties such as charge and hydration. In ribosomal protein S6, cooperative folding can still occur even after most of its surface charges are removed (via mutagenesis and pH control), yet the protein becomes markedly more prone to aggregation in both folded and denatured states [71], as illustrated in Figure 4A. This demonstrates that charge can play independent roles in intramolecular folding and intermolecular aggregation. Accordingly, charged residues are often referred to as aggregation gatekeepers [68,71,72]. In a cis-acting manner, such charged residues can protect aggregation-prone regions within the same polypeptide by generating intermolecular repulsive forces, without requiring attractive interactions. The aggregation gatekeeper concept can therefore be extended, in principle, to macromolecular tethering at larger scales (Figure 4B). Ribosomes display extensive negative surface charges. It is plausible that these surface charges collectively contribute to ribosome solubility, analogous to the role of charged residues as aggregation gatekeepers in S6. While the contribution of ribosomal surface charges to ribosome solubility is generally accepted, their implications for the aggregation of ribosome-tethered nascent chains have not been explicitly considered. Because nascent chains remain physically tethered to ribosomes, aggregation driven by these chains is expected to be sensitive to the electrostatic properties of the ribosomal surface that modulate intermolecular interactions. This scaling-based reasoning is qualitatively consistent with classical DLVO (Derjaguin–Landau–Verwey–Overbeek) theory, in which the electrostatic repulsive potential between charged entities can be approximated as $U_{\mathrm{rep}}(r)\;\propto \;Z_{1}Z_{2}\;\lambda_{B}\;\frac{e^{-\kappa r}}{r},$ where $Z_{1}$ and $Z_{2}$ denote the effective charges of the interacting entities, $\lambda_{B}$ is the Bjerrum length, and $\kappa^{-1}$ is the Debye screening length [73]. In this formulation, electrostatic repulsion scales with the product $Z_{1}Z_{2}$, suggesting that the large net surface charge of ribosomes may modulate not only ribosome solubility but also the aggregation of tethered nascent chains. Consistently, tethering proteins to highly soluble carriers has been shown to suppress aggregation [74,75,76].

By definition, excluded volume refers to a spatial region that cannot be occupied by other molecules at the same time [77,78]. As a result, aggregation of nascent chains—including their folding and native intermolecular interactions—proceeds under excluded-volume constraints imposed by ribosomes. When the excluded volume is defined by ribosome-scale dimensions (~20–30 nm in diameter), these constraints can extend the spatial scale over which steric exclusion operates in intermolecular encounters relevant to protein aggregation. Accordingly, aggregation of nascent chains may be significantly limited by the excluded volume imposed by ribosomes. Importantly, pure excluded-volume effects are independent of a molecule’s surface chemistry. Accordingly, to maintain solubility in aqueous environments, biological and synthetic macromolecules typically present ionizable charges or hydrophilic groups on their surfaces. A more comprehensive understanding of macromolecular repulsions therefore requires integrating both steric and surface-derived repulsive interactions. Notably, the steric repulsion-mediated aggregation inhibition described here differs from the conventional excluded-volume effect, even though both arise from the same excluded-volume constraint. In the conventional view, macromolecules act as inert crowders that reduce available volume, thereby entropically biasing systems toward the formation of more compact states, including folded conformations and aggregates [77,79].

Physical association of a polypeptide with a cellular macromolecule effectively increases its hydrodynamic size. As a result, according to Stokes–Einstein-type relations (and Stokes–Einstein–Debye-type relations for rotation), both translational diffusion and rotational tumbling are reduced, leading to lower translational and rotational mobility than a freely diffusing chain [80,81]. This behavior follows directly from hydrodynamic size scaling. Experimentally, nascent polypeptide chains tethered to ribosomes display markedly slower rotational dynamics, and membrane-anchored proteins diffuse substantially more slowly [82,83]. Together, reduced diffusion and rotational mobility are expected to limit diffusion-limited aggregation by decreasing the frequency of intermolecular encounters and the likelihood of productive intermolecular collisions.

**Figure 5:**
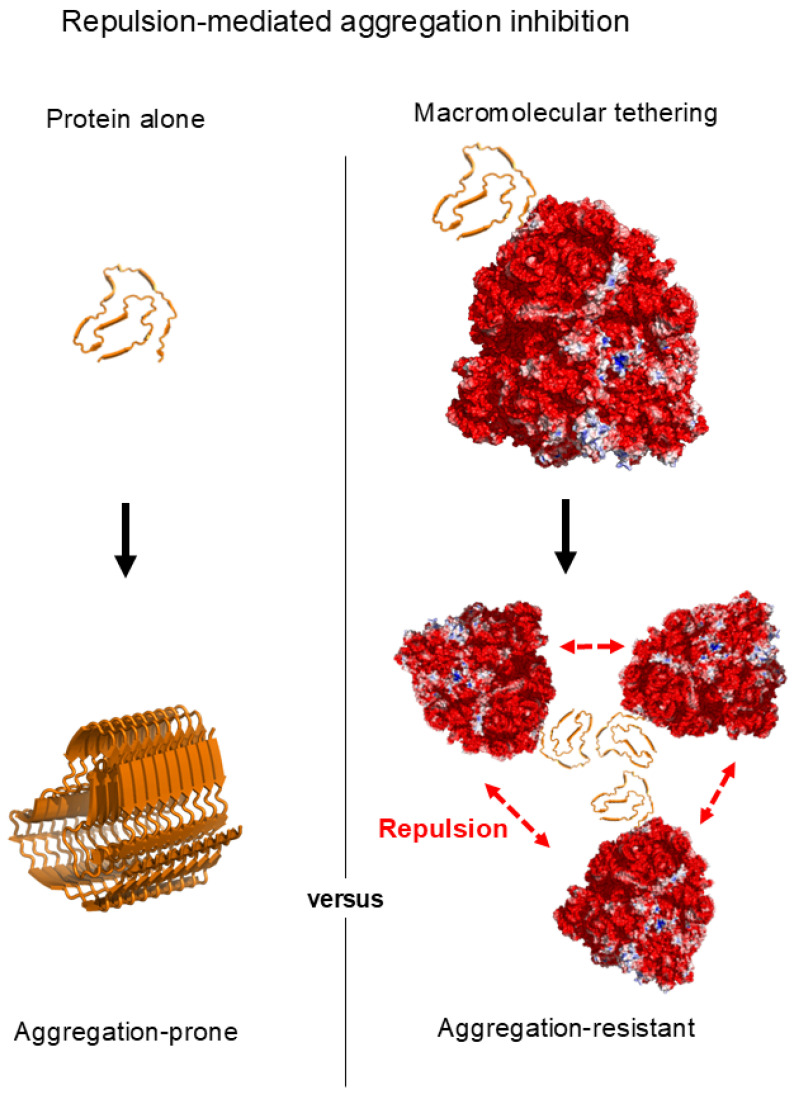
Comparison of aggregation between a freely diffusing state (**left**) and a tethered state (**right**). In the tethered context, intermolecular repulsive forces arising from excluded volume and surface charges of cellular macromolecules, such as ribosomes, keep proteins in an aggregation-resistant state.

Taken together, repulsions arising from the surface charges and excluded volume of cellular macromolecules can act in concert to limit aggregation of their tethered polypeptides, thereby promoting productive folding (Figure 5) [22,23]. In this sense, the aggregation gatekeeper concept extends from charged residues in polypeptides to cellular macromolecules via macromolecular tethering. In the tethered context, proteins can fold according to their intrinsic conformational preferences while intermolecular aggregation is constrained by steric and electrostatic repulsions operating over larger spatial scales. This protection does not require direct attractive interactions with aggregation-prone regions. Rather, it emerges from the physical properties of the attached macromolecule and can operate whenever physical linkage is established, whether in cis or in trans. Collectively, these mechanisms of repulsion-mediated aggregation inhibition, together with the scaling principle, suggest that cellular macromolecules can exhibit a generic intrinsic chaperone-like activity arising directly from their physicochemical properties.

### 3.2. Repulsion-Based Interpretation of Molecular Chaperone Action

Aggregation inhibition by molecular chaperones can likewise be understood in terms of the intermolecular repulsive forces arising from their excluded volume and surface charges, as in other cellular macromolecules. In this section, we consider how this physical logic provides a shared yet complementary basis for understanding certain aspects of the actions of GroEL/ES, Hsp70, and Hsp90. Specific binding interactions in classical chaperone systems play central roles in substrate recognition and can modulate folding and aggregation, whereas repulsion-mediated effects represent an additional mechanism for suppressing intermolecular aggregation. These mechanisms are not mutually exclusive and can operate simultaneously.

**Figure 6:**
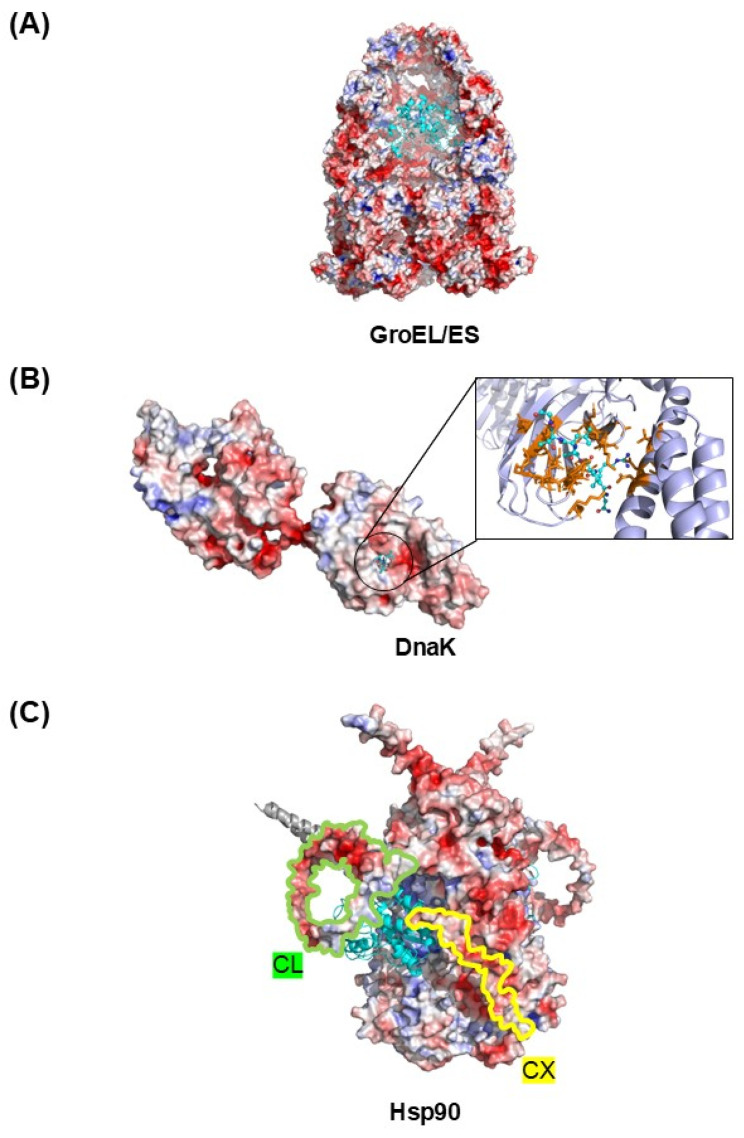
Repulsion-based interpretation of molecular chaperone action. (**A**) GroEL/ES encapsulates client proteins within a confined cavity, where steric exclusion limits intermolecular aggregation. (**B**) DnaK recognizes exposed peptide segments (e.g., NRLLLTG) and binds them within a substrate-binding groove; the hydrophobic contacts involved in recognition are relatively limited. (**C**) Hsp90 contains highly charged regions, including the acidic linker (CL) and the C-terminal extension (CX), whose surface charges contribute to its anti-aggregation activity. In all panels, client proteins and peptide segments are shown in cyan.

The three-dimensional architecture of GroEL/ES provides a structural basis for aggregation inhibition imposed by excluded-volume constraints (Figure 6A) [84]. The GroEL/ES chamber is called the Anfinsen cage [85]. This chamber accommodates proteins up to ~60 kDa [86], a size limitation that itself reflects geometric excluded-volume constraints imposed by the cavity. Substrates exceeding this range, such as aconitase (~82 kDa), can nonetheless fold productively through repeated binding–release cycles with GroEL without complete encapsulation [87]. Even without full encapsulation, association with the surface of GroEL can impose steric and electrostatic repulsive effects that limit aggregation of the associated polypeptide. With respect to folding, encapsulation can accelerate folding for some substrates, consistent with confinement theory and excluded-volume effects on the accessible conformational ensemble [86,88]. However, the GroEL/GroES cavity can also be strongly destabilizing for at least some substrates; in a model substrate, encapsulation reduced stability by >5 kcal mol^−1^ relative to bulk solution, strongly favoring the unfolded state [89]. This illustrates that confinement does not universally stabilize folded states. If a protein’s native conformations are sterically incompatible with the imposed excluded-volume constraints, confinement can shift the conformational equilibrium toward partially unfolded or destabilized states. Similarly, proteins confined within narrow ribosomal exit tunnels or membrane pores adopt restricted conformations, such as α-helices, because only structures compatible with the steric constraints can be accommodated.

DnaK, an *E. coli* Hsp70 homolog, binds peptide segments (e.g., NRLLLTG) in an extended conformation in a channel or groove formed by the β-subdomain, with a helical lid region stabilizing the bound state [90], as shown in Figure 6B. DnaK preferentially recognizes and binds hydrophobic core segments of ~4–5 residues, often flanked by basic residues, which occur approximately once every 30–40 residues in client proteins [91]. Although such limited hydrophobic interactions are sufficient for substrate recognition, they are unlikely to fully account for DnaK’s stabilizing effect. To separate the roles of substrate recognition and stabilization, DnaK–substrate interactions were converted from a trans-acting system to a cis-linked system by covalently fusing DnaK to the N-terminus of aggregation-prone proteins in vivo. In this fusion context, DnaK efficiently solubilized the linked proteins, even when the C-terminal substrate-binding domain was deleted [62]. This indicates that, once linkage is provided by covalent tethering, at least part of DnaK’s anti-aggregation activity may be independent of the canonical substrate-binding domain. Thus, intermolecular repulsions arising from excluded volume and surface charges of DnaK have been hypothesized to contribute to substrate stabilization against aggregation. Excluded-volume constraints by Hsp70 can give rise to entropic pulling forces. These forces have been proposed as a unifying physical mechanism for several active Hsp70 functions, including protein translocation, unfolding, and disaggregation [92]. When Hsp70 is positioned near a membrane or a translocation pore, its configurational freedom is restricted by steric constraints. Moving the Hsp70–substrate complex away from this region increases entropy, thereby generating an entropic bias that favors directional motion. Nanopore single-molecule experiments provide quantitative support for this mechanism. Binding of DnaK to a polypeptide confined within a pore promoted extraction of the trapped chain, with an associated free-energy change of ~10–12 k_B_T, corresponding to an effective force of ~40–50 pN over 1 nm [93]. Similarly, excluded-volume repulsions between multiple DnaK molecules bound to a single client protein can destabilize or melt compact misfolded intermediates, thereby increasing refolding rate [13,94]. Overall, these observations indicate that the generic physicochemical properties of Hsp70 can contribute not only to substrate stabilization but also to Hsp70′s active functions.

Hsp90 contains highly charged regions, most prominently the acidic, charged linker (CL) located between the N-terminal and middle domains and the C-terminal extension (CX) (Figure 6C). Simultaneous deletion of both regions (ΔCLΔCX) markedly impairs Hsp90′s ability to suppress aggregation of misfolded substrates such as CFTR-NBD1 (cystic fibrosis transmembrane conductance regulator nucleotide-binding domain 1) and citrate synthase, whereas individual deletions have minimal effects [95]. Appending the CL sequence to the C-terminus of the ΔCLΔCX construct restores anti-aggregation activity, demonstrating that net negative charge contributes to this activity independently of sequence-specific binding interactions [95]. A similar principle applies to αB-crystallin: its flexible, polar/charged C-terminal extension helps maintain the solubility of both the chaperone and chaperone–client complexes [96]. Moreover, plant stress chaperones such as dehydrins function through electrostatic and steric mechanisms: Lys-rich segments promote weak, salt-sensitive proximity to client surfaces without stable binding, while the disordered scaffold provides steric and entropic shielding that suppresses aggregation in a size-dependent manner [97,98]. In addition, charged surfaces in molecular chaperones can contribute to substrate recognition by promoting long-range electrostatic steering toward aggregation-prone clients, as shown for chaperones such as GroEL, TRiC/CCT, Trigger Factor, Spy, and DAXX [99,100,101,102,103]. Electrostatic interaction-mediated substrate recognition has been described as distinct from canonical hydrophobic interaction-driven recognition [101]. Repulsion-mediated aggregation inhibition, however, can operate independently of the mode of substrate recognition—whether through hydrophobic or nonhydrophobic interactions—thereby accommodating diverse interaction types.

## 4. Cis-Acting Chaperoning by Macromolecular Tethering

In this section, we discuss three representative forms of macromolecular tethering—ribosome tethering, domain tethering, and membrane tethering—in the context of cis-acting chaperoning. In particular, the independent effects of macromolecular tethering on folding and aggregation are central to understanding cis-acting chaperoning in tethered systems. Under tethered conditions, proteins can fold according to their intrinsic, sequence-encoded properties while intermolecular aggregation is suppressed.

### 4.1. Ribosome Tethering

During translation, nascent polypeptides are tethered to ribosomes in cis via a covalent peptidyl–tRNA linkage. Before productive folding occurs, cytosol-exposed nascent chains emerging from polysomes have traditionally been regarded as highly prone to aggregation, owing to their close spatial proximity and high local concentration [5]. This presumed aggregation risk at the ribosomal exit site has been used to justify early engagement by ribosome-associated chaperones such as Trigger Factor and Hsp70, reinforcing the view that molecular chaperones assist de novo folding rather than acting solely in refolding or stress-recovery pathways [5,104,105,106,107]. However, this view does not explicitly consider the effect of ribosome tethering on aggregation.

The following examples demonstrate that ribosome-tethered nascent polypeptides can adopt aggregation-resistant, folding-competent states. In ribosome display systems that preserve the native ribosome–nascent chain complex, highly aggregation-prone receptor domains remain soluble and functional when tethered to ribosomes, but aggregate rapidly upon release from the ribosome [108]. Similarly, several aggregation-prone proteins that form inclusion bodies when expressed alone in the *E. coli* cytoplasm remain soluble and functional when covalently tethered to ribosomes via fusion to ribosomal protein L23 [109]. Consistent with this view, immobilization of the C-terminus of luciferase on column beads increases refolding yield by suppressing aggregation, an effect proposed to be physically analogous to ribosome tethering during cotranslational folding [110]. Cryo–electron tomography shows that bacterial polysomes adopt a staggered, pseudohelical architecture that spatially separates ribosome-tethered nascent chains, thereby reducing aggregation-prone interchain interactions [111]. In addition, RNAs can suppress aggregation and enhance folding yield when bound to their cognate RNA-binding modules fused to aggregation-prone proteins, suggesting an intrinsic chaperone-like activity of RNAs [112]. Such RNA–protein complex-tethered aggregation-prone proteins physically resemble ribosome-tethered nascent chains, supporting cis-acting chaperoning by ribosomes and their associated rRNAs [113].

In contrast to classical in vitro refolding, which largely reflects post-translational folding, vectorial protein synthesis on ribosomes provides a physical context that enables cotranslational folding. Experimental studies using advanced techniques have shown that nascent polypeptides can form secondary-structure elements and subdomain-level folding intermediates while tethered to ribosomes [114,115,116]. Electrostatic interactions between the ribosome and the nascent polypeptide influence this process: the highly negatively charged ribosomal surface and exit tunnel can interact with emerging positively charged segments of the nascent chain, tending to destabilize compact partially folded structures and maintain the polypeptide in a more dynamic, flexible state [117,118,119]. This can delay the acquisition of a stable fold until sufficient chain length has emerged, thereby reducing the risk of premature folding, misfolding, or kinetic trapping. In specific cases, however, the ribosome can also stabilize particular folding intermediates, reshaping the folding energy landscape [120]. The negatively charged and confined ribosome exit tunnel has been suggested to provide a folding environment reminiscent of the negatively charged surfaces of the GroEL/ES folding cage [1]. For small single-domain proteins that fold cooperatively in a two-state manner, ribosome tethering does not fundamentally alter the intrinsic folding mechanism [121]. By contrast, multidomain proteins often fold sequentially during translation, domain by domain; cotranslational folding helps minimize inter-domain misfolding and aggregation [122].

Accordingly, ribosome tethering allows cotranslational folding to proceed largely according to the intrinsic properties of proteins while effectively mitigating aggregation. This cis-acting chaperoning by ribosome tethering constitutes a built-in mode of chaperoning that is inherently coupled to protein synthesis. Although ribosome tethering is generally protective against aggregation, it should not be regarded as absolute, as the close spatial proximity of nascent chains in polysomes may, under certain conditions, facilitate nonnative intermolecular interactions.

### 4.2. Domain Tethering

Multidomain proteins are often characterized by rugged folding energy landscapes and a high propensity for misfolding and aggregation, as suggested primarily by in vitro refolding experiments and simulations [123]. In vivo, domain-wise cotranslational folding allows upstream domains to fold before downstream domains are fully synthesized, creating a folding context distinct from that observed during in vitro refolding of full-length proteins. Yet, the effects of pre-folded (or cotranslationally folded) domains on both the folding and aggregation of their tethered neighboring domains remain largely unexplored. Moreover, native interdomain interactions mediated by cis linkages fall outside the conventional definition of molecular chaperones. Insights into the potential cis-acting chaperoning of such domains can be obtained from fusion-based approaches widely used in biotechnology.

Fusion strategies, in which aggregation-prone heterologous proteins are covalently linked to the C-termini of soluble partners, are widely used in *E. coli* as a robust method to suppress aggregation and enhance folding efficiency [47,124,125,126]. A broad range of fusion partners, including maltose-binding protein (MBP), thioredoxin, glutathione S-transferase, and NusA, have been employed for this purpose [47,127,128,129]. In addition, short peptide tags enriched in charged residues have also been shown to improve solubility and folding yield [76]. In the fusion context, solubility tags such as MBP and NusA are often described as “chaperone-like” or “passive” solubilizers, in the sense that they can keep aggregation-prone cargo proteins soluble while they either fold spontaneously or are folded by endogenous chaperone systems [130,131]. Nonetheless, fusion strategies have generally been viewed as technical tools for the production of functional proteins, and their potential physiological relevance has largely remained unexplored in protein science. These consistent observations suggest that soluble fusion partners may possess intrinsic chaperone-like activity that operates even when physically linked to their cargo proteins via simple, flexible linkers. Viewed in this light, artificial fusion proteins can be considered multidomain proteins in which the N-terminal fusion partner exerts a chaperone-like influence on the downstream cargo domain—raising the possibility that similar cis-acting effects operate in native multidomain proteins. To explore this possibility, N-terminal domains from native *E. coli* proteins, such as lysyl-tRNA synthetase (LysRS), threonyl-tRNA synthetase, and aconitase B, have been fused to diverse aggregation-prone heterologous proteins, resulting in markedly improved solubility and folding yield [22]. Because this approach eliminates native interdomain interactions, it enables the intrinsic chaperone-like activity of individual domains to be assessed in isolation, although such fusion constructs do not fully recapitulate their native multidomain proteins. Many domains of native multidomain proteins have been shown to function as solubility-enhancing tags for heterologous proteins [22,62,132,133,134,135,136,137,138,139,140,141,142], as summarized in Table 1. Collectively, these findings suggest that, despite the artificial nature of fusion constructs, folded domains derived from native multidomain proteins can exhibit intrinsic chaperone-like activity in cis, supporting the view that cis-acting chaperoning observed in biotechnology reflects a physiologically relevant phenomenon. Moreover, the chaperone-like activity of these domains correlates with their surface charge and molecular size, consistent with a model in which electrostatic and steric repulsions exerted by folded domains suppress aggregation of linked polypeptides and thereby promote productive folding [22]. Supporting this view, highly charged intrinsically disordered proteins function as solubility-enhancing tags by acting as entropic bristles that prevent aggregation through electrostatic repulsion, steric exclusion, and enhanced solvation [143]. Similarly, the disordered, negatively charged N-terminal tails of SUMO proteins act as intramolecular entropic bristles that suppress aggregation of the SUMO globular domain by steric and electrostatic repulsion, without altering its native structure or function [144].

Independently of aggregation, domain tethering can influence the folding of adjacent domains. When a folded domain engages in native interdomain interactions with a neighboring domain, it can enhance folding by stabilizing the transition-state ensemble (thereby accelerating folding) or by stabilizing the native state (reducing the unfolding rate). Studies of artificial fusion constructs show that a folded but nonnative tethered fluorescent protein can destabilize its fused partner domain [145,146]. This destabilization has been proposed to arise from entropic stabilization of the denatured-state ensemble of the tethered protein caused by the presence of the large folded green fluorescent protein [145]. Folded domains can influence the folding rates or thermodynamic stability of neighboring domains. However, extensive Φ-value analyses of native multidomain proteins, together with studies of artificial multidomain proteins, show that the presence of a folded neighboring domain does not alter the intrinsic folding mechanism of the adjacent domain [147]. Within the folding-centric framework, this thermodynamic destabilization by fluorescent protein tagging would be expected to promote aggregation; however, given the independent effects of this tagging on intramolecular folding and intermolecular aggregation, the final folding yield and solubility of the tagged proteins may increase. These discrepancies between folding and aggregation are not exceptional but rather ubiquitous [18], as emphasized throughout this paper.

Overall, these findings indicate that folded domains can exert cis-acting chaperoning, whose contribution to aggregation suppression cannot be fully explained by changes in folding kinetics, stability, or mechanism alone because domain tethering can influence folding and aggregation independently. In addition to cotranslational folding, molecular chaperones, and native interdomain interactions, this cis-acting chaperoning by folded domains may represent another built-in layer of cellular chaperoning that enables large multidomain proteins to remain soluble and fold productively.

### 4.3. Membrane Tethering

Membrane tethering in cis is mediated either by hydrophobic transmembrane domains that insert into the lipid bilayer or by covalent lipid anchors generated by lipidation, such as glycosylphosphatidylinositol (GPI) anchoring, N-terminal myristoylation, S-palmitoylation, and prenylation [148]. Secreted proteins are physically and topologically constrained by membrane-associated targeting and translocation machinery (e.g., ribosome–translocon engagement at the ER) before being released into their final extracellular or luminal environments. Such tethering imposes spatial and topological constraints on the polypeptide, thereby modulating its folding and aggregation behavior.

Both in vitro and cell-based studies show that anchoring aggregation-prone polypeptides to membranes markedly reduces their propensity to form pathogenic amyloids. For example, the cellular prion protein, which is tethered to the cell surface via a GPI anchor, remains in a stable native state, whereas altered membrane attachment or mislocalization can promote its conversion into amyloid aggregates [149,150]. Similarly, yeast prion protein Sup35 and Alzheimer’s Aβ peptide remain non-amyloidogenic while associated with membranes or membrane-bound precursors but readily aggregate upon release. Serum amyloid A remains soluble when bound to lipid particles such as high-density lipoproteins, yet rapidly forms amyloid fibrils once dissociated into the aqueous phase [151]. These principles have been exploited in biotechnology, where membrane tethering is used to improve soluble, functional expression of aggregation-prone recombinant proteins. One example is anchored periplasmic expression (APEx) in *E. coli*, which displays proteins tethered on the periplasmic face of the inner membrane [152]. Beyond covalent anchors, reversible membrane interactions can also modulate protein aggregation. α-Synuclein transiently binds lipid bilayers via an N-terminal amphipathic helix, and lipid binding can inhibit fibril formation under conditions where membrane association stabilizes non-amyloidogenic conformations [153,154,155]. However, membrane surfaces can also promote amyloid nucleation under specific conditions (e.g., low lipid-to-protein ratios), highlighting the context-dependent nature of membrane effects on aggregation [156]. This effect is likely influenced by multiple factors involved in aggregation formation, including increased local concentration on a two-dimensional surface and binding-induced conformational changes, which may facilitate the nucleation step.

Membrane proteins fold in a physicochemical environment fundamentally distinct from that of proteins in aqueous solution. Integral membrane proteins must accommodate the hydrophobic interior of lipid bilayers, where exposure of polar backbone groups to the hydrophobic core is energetically unfavorable. Consequently, membrane-spanning segments typically adopt regular secondary structures—α-helices or β-sheets assembled into β-barrels—in which backbone amide and carbonyl groups are internally hydrogen-bonded [157,158]. By contrast, extramembrane domains exposed to the aqueous environment on either side of the membrane generally follow folding principles similar to those of soluble proteins [159,160]. Nevertheless, folding and assembly of full-length membrane proteins—including insertion, topology, and quality control of hydrophobic segments—often require cotranslational insertion and specialized membrane-protein biogenesis factors and chaperones [157,158].

Collectively, these observations indicate that membrane tethering can suppress aggregation of membrane-anchored proteins. Similar aggregation-limiting effects may also occur for peripheral proteins upon membrane binding, as the complexed state imposes spatial constraints regardless of whether the association occurs in cis or in trans.

## 5. Interconvertibility of Cis- and Trans-Acting Chaperoning

**Figure 7:**
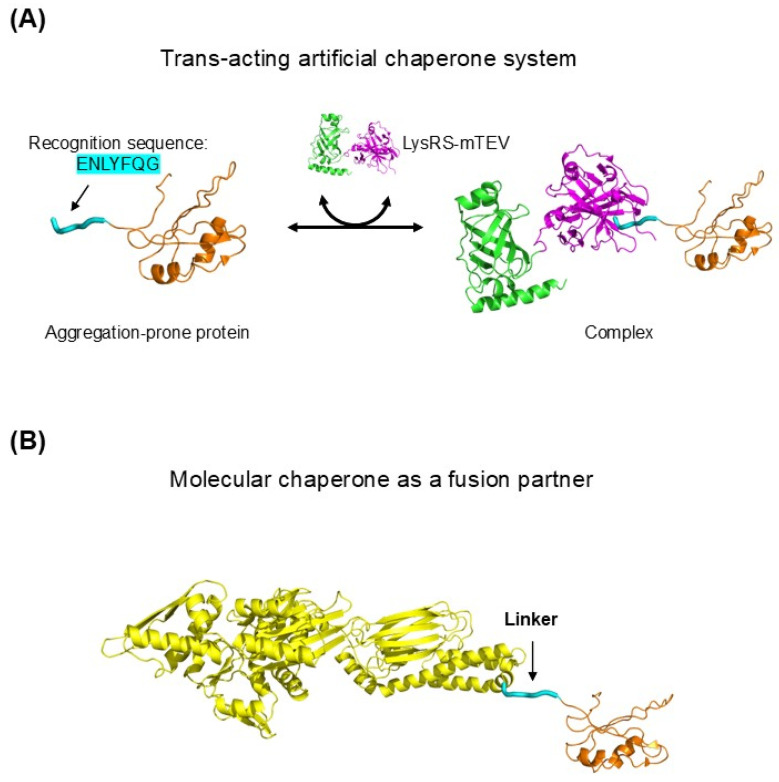
Interconversion of cis- and trans-acting chaperoning. (**A**) Engineered trans-acting system in which LysRS–mTEV binds aggregation-prone proteins via the TEV protease recognition sequence (ENLYFQG), shown in cyan. (**B**) Fusion of molecular chaperones, such as DnaK (shown in yellow), to aggregation-prone proteins illustrates cis-acting chaperoning. The linker region is shown in cyan.

The intrinsic chaperone-like activity of cellular macromolecules, supported by the observed cis-acting chaperoning and repulsion-mediated aggregation inhibition, suggests interconvertibility between cis- and trans-acting chaperoning. LysRS–mTEV, a soluble recombinant protein, can be converted into a trans-acting chaperone for proteins containing its recognition sequence (ENLYFQG) (Figure 7A). This artificial chaperone functions independently of whether the recognition tag is located at the N-terminus, C-terminus, or within the internal linker region of the substrate [161]. Moreover, it exhibits little or no chaperone-like activity under non-aggregating conditions in vitro, indicating that it assists protein folding indirectly by preventing aggregation. This artificial trans-acting chaperone system was designed to recapitulate cis-acting chaperoning by macromolecular tethering: the covalent linkage in cis is replaced functionally by a specific trans-acting association mediated by the recognition tag. LysRS exhibits strong chaperone-like activity when directly fused to diverse aggregation-prone proteins, similar to conventional solubility-enhancing fusion partners [112]. Notably, the chaperone activity of LysRS–mTEV is largely attributable to the highly soluble LysRS moiety, which does not directly bind substrates, whereas mTEV alone shows little or no chaperone activity due to its limited solubility. These observations indicate that LysRS can exert chaperone-like activity through both cis and trans modes of linkage. Likewise, a wide range of trans-acting molecular chaperones and folding catalysts—including DnaK, GroEL, Trigger Factor, Spy, PDI, and PPI—have been used as fusion partners to enhance the solubility and folding yield of aggregation-prone proteins in *E. coli* [162,163,164,165,166], as illustrated in Figure 7B. In this fusion context, cis-acting chaperoning by molecular chaperones likely operates through mechanisms similar to those of conventional solubility-enhancing fusion partners. This interconvertibility between cis- and trans-acting chaperoning is consistent with the view that intrinsic chaperone-like activity arises from the generic physicochemical properties of cellular macromolecules, most plausibly repulsion-mediated aggregation inhibition.

A characteristic feature of the cellular milieu is that, throughout their lifetimes, proteins are physically linked—either in cis or in trans—to a variety of cellular macromolecules. In addition to the forms of macromolecular tethering discussed here, a large fraction of proteins in animal cells undergo covalent modifications, most prominently by ubiquitin and also by SUMO (∼15–20%) or glycans (∼50%) during their life cycles [167,168,169]. Given this pervasive physical linkage, the intrinsic chaperone-like activity of cellular macromolecules, together with repulsion-mediated aggregation inhibition, offers a broader perspective on cellular chaperoning.

## 6. Conclusions

We describe cis-acting chaperoning by macromolecular tethering, whereby ribosomes, lipid bilayers, and folded domains suppress aggregation of tethered polypeptides and thereby promote productive folding. By systematically addressing conceptual gaps in prevailing frameworks of protein science—including trans-acting interaction-centric, folding-centric, and structure-formation-centric frameworks—this work clarifies why cis-acting aggregation control has remained largely underrecognized. Our analysis further establishes a conceptual bridge between cis- and trans-acting chaperoning by highlighting repulsion-mediated aggregation inhibition as a shared physicochemical principle analogous to colloidal stability. Consistent with this principle, ribosome tethering, domain tethering, and membrane tethering represent forms of cis-acting chaperoning that function analogously to scaled-up aggregation gatekeepers, constituting a built-in layer of cellular chaperoning. The interconvertibility between cis- and trans-acting chaperoning further supports the view that intrinsic chaperone-like activity arises from the generic physicochemical properties of cellular macromolecules. Together, these insights expand the conceptual scope of cellular chaperoning and provide a framework for understanding how macromolecular tethering contributes to proteome solubility and productive folding in the cellular environment.

## Figures and Tables

**Table 1:** Native protein domains used as solubility-enhancing fusion partners. Representative domains from multidomain proteins used as N-terminal tags to enhance the solubility and folding yield of aggregation-prone proteins in *E. coli*, including domains from proteins of diverse sizes and origins.

Multidomain Proteins	Domain (aa)	Domain’s Size (kDa)	Origin	References
Lysyl-tRNA synthetase	1–154	16.9	*E. coli*	[22]
Threonyl-tRNA synthetase	1–225	24.8	*E. coli*	[22]
Aconitase B	1–160	17.6	*E. coli*	[22]
DnaK	1–384	42.2	*E. coli*	[62]
GroEL	191–345	17.1	*E. coli*	[132]
Protein disulfide isomerase	214–440	25	Human	[133]
Protein G	1–56	6.2	*Streptococcus* sp.	[134]
Translation IF2	1–158	17.4	*E. coli*	[135]
RpoD (σ70)	33–215	20.1	*E. coli*	[136]
PTS enzyme I	1–249	27.4	*E. coli*	[137]
TolA	314–421	10.8	*E. coli*	[138]
Pyruvate dehydrogenase E2	1–33 + 238–289	9.3	*E. coli*	[139]
Spidroin	1–130	14.3	*Spider* (*E. australis* MaSp1)	[140]
Endoglucanase-xylanase D	349–487	15.3	*Clostridium cellulovorans*	[141]
Exo-levanase (BsSacC)	514–677	18.0	*Bacillus subtilis*	[142]

## Data Availability

No new data were created or analyzed in this study. Data sharing is not applicable to this article.
